# Genome-wide Annotation and Comparative Analysis of Long Terminal Repeat Retrotransposons between Pear Species of *P. bretschneideri* and *P. Communis*

**DOI:** 10.1038/srep17644

**Published:** 2015-12-03

**Authors:** Hao Yin, Jianchang Du, Jun Wu, Shuwei Wei, Yingxiu Xu, Shutian Tao, Juyou Wu, Shaoling Zhang

**Affiliations:** 1 Center of Pear Engineering Technology Research, State Key Laboratory of Crop Genetics and Germplasm Enhancement, Nanjing Agricultural University, Nanjing 210095, Jiangsu, China; 2 Institute of Biotechnology, Jiangsu Academy of Agricultural Sciences, Nanjing 210014, Jiangsu, China

## Abstract

Recent sequencing of the Oriental pear (*P. bretschneideri* Rehd.) genome and the availability of the draft genome sequence of Occidental pear (*P. communis* L.), has provided a good opportunity to characterize the abundance, distribution, timing, and evolution of long terminal repeat retrotransposons (LTR-RTs) in these two important fruit plants. Here, a total of 7247 LTR-RTs, which can be classified into 148 families, have been identified in the assembled Oriental pear genome. Unlike in other plant genomes, approximately 90% of these elements were found to be randomly distributed along the pear chromosomes. Further analysis revealed that the amplification timeframe of elements varies dramatically in different families, super-families and lineages, and the *Copia*-like elements have highest activity in the recent 0.5 million years (Mys). The data also showed that two genomes evolved with similar evolutionary rates after their split from the common ancestor ~0.77–1.66 million years ago (Mya). Overall, the data provided here will be a valuable resource for further investigating the impact of transposable elements on gene structure, expression, and epigenetic modification in the pear genomes.

Retrotransposons are abundant and widespread mobile DNA in eukaryotic genomes. It has been documented that long terminal repeat retrotransposons (LTR-RTs) are particularly common in flowering plants^1^. Comprehensive analyses from several assembled plant genomes has provided evidence that many genomes, such as 19% of peach^2^, 62% of tomato^3^, 53% of potato^4^, and over 70% of maize genomes^5^, are composed of LTR-RTs.

LTR-RTs can be classified into different super-families and families based on the structures and the sequence identities between elements^6^. A representative autonomous intact LTR-RT is comprised of two identical or similar LTRs, 4–6-bp target site duplication (TSD) flanking with its 5′ and 3′ ends, a primer-binding site (PBS), a polypurine tract (PPT), and two functional genes (*gag*, and *pol*) (Kumar and Bennetzen 1999). Based on the order of *rt* and *int* in *pol*, LTR-RTs can be further classed into *Gypsy* and *Copia* super-families^7^. In addition, the LTR-RTs also contain two specific groups, *la*rge *r*etrotransposon *d*erivatives (LARDs)^8^ and *t*erminal-repeat *r*etrotransposons *i*n *m*iniature (TRIMs)^9^. In the LARD elements, the coding region is replaced by a large conserved noncoding DNA sequence (usually >4 kb) whereas in TRIM elements, the internal part between two LTRs is very short and thus the whole element is very small. Based on the coverage and sequence identities, LTR-RTs can be separated into different families^6^. For example, the 32,370 soybean LTR-RT elements have been classified into 510 distinct families^10^, and 526 intact LTR-RTs from *Medicago truncatula* have been separated into 85 individual families^11^.

*Pyrus* (pear) belongs to the tribe *Pyrinae*, super-tribe *Pyreae* in the *Spiraeoideae* subfamily of Rosaceae^12^, and is one of the most economically important fruit crops in the temperate zones, cultivated in more than 50 countries^13^. Because of the high diversity of agronomic traits, such as fruit shape^14^, fruit aroma^15^, and disease resistance^16^, *Pyrus* species have drawn tremendous attention, and their origins and relationships have been widely studied^17^,^18^,^19^,^20^. According to the geographic distribution, the *Pyrus* species can be traditionally divided into two native groups: Occidental pears and Oriental pears^21^. *P. communis* L., an Occidental pear, is the most commonly cultivated pear species in Europe, North America, South America, Australia, and Africa. Occidental pears have been cultivated in Europe since as early as 1000 BC, and ‘Bartlett’ is the most important cultivar worldwide. Conversely from the single cultivated species *P. communis* L. in Occidental pears, the Oriental pears can be separated into four species, *P. pyrifolia* Nakai., *P. ussuriensis* Maxim., *P.* × *bretschneideri* Rehd., and *P.* × *sinkiangensis* Yu.^17^. The three *P. bretschneideri* cultivars, including ‘Dongshansuli’, ‘Yali’ and ‘Huanghuali’, have made China the world’s leader in Oriental pear production.

The availability of the draft genome sequences of the Oriental pear (*P. bretschneideri* Rehd.) ‘Dangshansuli’^22^ and Occidental pear (*P. communis* L.) ‘Bartlett’^23^ provides us unprecedented opportunities for comparative analysis of LTR-RT elements, evolutionary history, and the divergence process in these important Rosaceae species. Previously, only one *Copia*-type LTR-RT family, *Ppcrt*, had been identified using 454 sequencing data in Japanese pear (*Pyrus pyrifolia*) ‘Hosui’, and retrotransposon-based insertional polymorphism (RBIP) markers have been developed for DNA profiling of 80 pear cultivars^24^. In the present study, we first present the characterization of LTR-RTs in the pear genome, including structural analysis, distribution pattern, amplification timeframe, and lineage analysis of LTR-RTs. We have also analyzed the rates of nucleotide substitution between orthologous LTR-RTs, the rates of synonymous substitution (Ks) and non-synonymous substitution (Ka) between orthologous genes between the two *Pyrus* species genomes. Our data showed that more than 7,000 annotated LTR-RTs from the assembled genome sequence of Oriental pear (*P. bretschneideri* Rehd.) can be classified into 148 families. Overall, the elements exhibit an unbiased distribution along the chromosomes, while the *Copia*-like LTR-RTs are much more active within 1 Mys compared with other super-families. The *Copia*-like *Maximus* lineage has been lost in the Rosaceae species and the two pear species have evolved with similar evolutionary rates since they split from their common ancestor ~0.77–1.66 Mya. Thus this study, for the first time, reveals the abundance, distribution, and differential amplification of LTR-RTs, provides additional evidence supporting a previous study that nucleotide substitution rate of LTR-RTs is at least twofold over that of coding sequences, and uncovers the evolution patterns and divergence process between Oriental pear (*P. bretschneideri* Rehd.) and Occidental pear (*P. communis* L.) species.

## Results

### Identification and Structural Characterization of LTR-RTs in the Pear (*P. bretschneideri*) Genome

To accurately characterize the structure of LTR-RTs and their organization in the pear genome, we annotated LTR-RTs in the high-quality assembled Oriental pear (*P. bretschneideri*) genome based on previously used methods^10^,^25^, and particularly detailed manual inspections have been conducted to confirm each predicted element and define its structure and boundaries. In total, 7,247 elements with two clearly defined boundaries were identified. Truncated elements without structurally defined termini were not investigated in this study, because the present pear pseudo-chromosomes still contain numerous sequence gaps within and around repetitive sequences, and some truncated elements or fragments can potentially be products of incomplete assembly or mis-assembly. Of the 7,247 elements described above, 5,532 (76.3%) were anchored to the currently assembled 17 pseudo-chromosomes. Overall, 3,221 (44.4%) are intact elements with TSDs (IT), 578 (8.0%) are intact elements without TSDs (InT), 2,896 (40.0%) are solo LTRs with TSDs (ST), and 552 (7.6%) are solo LTRs without TSDs (SnT) (Table S1). Because it has been suggested that the InT and SnT elements were formed by inter-element unequal recombination between two adjacent LTR-RTs belonging to the same family, whereas the ST elements were formed by intra-element unequal recombination between the two intra-LTRs of the IT elements^26^. So the significant lower proportion of ST to IT in pear (0.90:1) than the ratio in rice (1.55:1) might indicate the pear has lower intra-element unequal recombination rate, compared with rice. In contrast, the higher proportions of InT and SnT to IT (0.35:1) in pear than in rice (0.23:1), are probably caused by the higher frequent inter-element unequal recombination in the former^25^.

**Figure 1:**
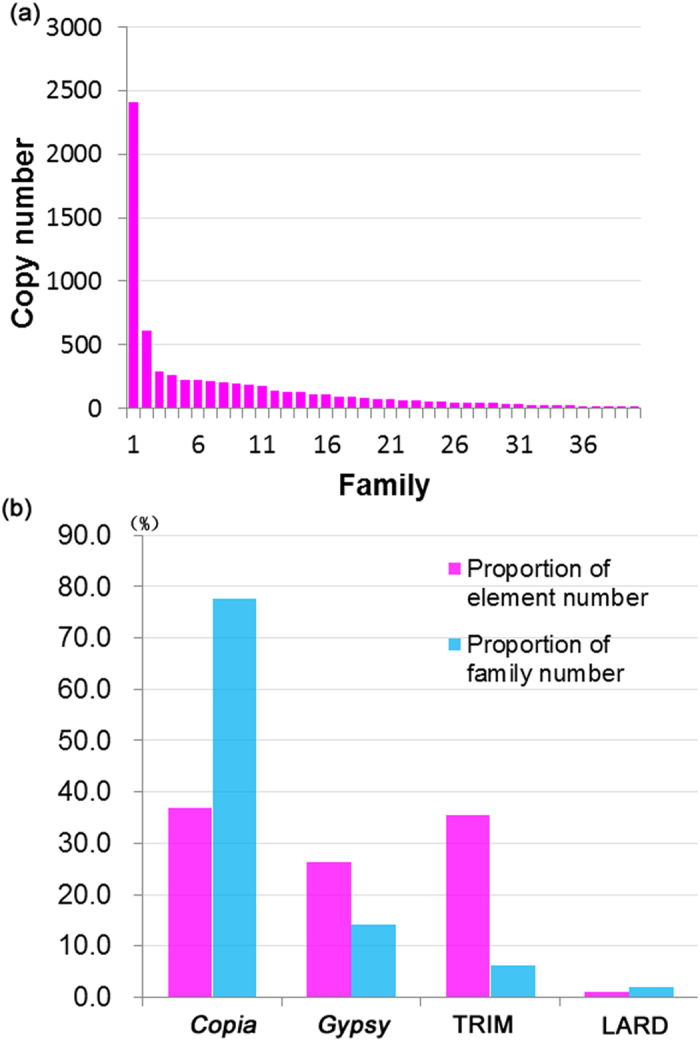
Variation of LTR-RTs copy number per families and super-families in *P. bretschneideri* genome. (**a**) *x* axis represents different families and *y* axis the copy number per family, only the top 40 families are presented. (**b**) *x* axis represents different lineages and *y* axis the proportion of copy number and family number per superfamily.

Based on the unified classification for eukaryotic transposable elements described by Wicker *et al.*^6^, the 7,247 elements were grouped into 148 distinct families, including 115 *Copia*-like families (2,675 elements), 21 *Gypsy*-like families (1,914 elements), 9 TRIM families (2,580 elements) and 3 LARD families (78 elements) (Fig. 1, Table 1). In the 148 families, only two families, *Ppcrt* and *PbrCassandra,* have been reported previously^24^,^27^, and the other 146 (98.6%) families were newly reported (Table S2). Overall, the IT, InT elements, and ST, SnT LTRs, together with numerous truncated fragments or remnants, in total make up 44.16% of the pear genome. This estimate is lower than estimated in the larger maize genome (79%)^5^ and sorghum genome (55%)^28^, but higher than the smaller rice genome (26%)^29^.

Despite the lower number of *Gypsy*-like elements (intact elements and solo LTRs), these elements and their related repetitive sequences make up more genomic DNA (25.5%) in pear than *Copia*-like elements do (16.9%). To explain this, the length variation of the two types of elements has been normalized first. The results showed that the average length of *Gypsy*-like elements (9.7-Kb) is about 1.53 fold of that of *Copia*-like elements (6.3-Kb), counterbalanced the variation of genomic DNA size of the two types elements (25.5/16.9 = 1.50). Thus, this could be explained by the older ages of *Gypsy*-like elements (also see below) and more truncated elements and related DNA fragments have been formed via frequent inter-element illegitimate recombination^26^. In addition to the above four types of elements, 252 ‘complex’ *PbrCassandra* elements with multiple LTRs (3, 4 and 5) have been identified^27^. These elements belong to the TRIM superfamily^9^,^30^, which were presumed to be produced by the inter-element unequal recombination followed by transposition^27^. Here we have found 5 other ‘LTR–internal–LTR–internal–LTR’ ‘complex’ elements belonging to *Pbr118* TRIM, including 4 with TSDs and 1 without TSD. Overall, the total number of ‘complex’ elements with multiple LTRs identified in pear (257) is much higher than that in rice (14)^25^, *Arabidopsis* (1)^26^, and Triticeae (2)^31^ genomes, but why so many ‘complex’ TRIM elements are present in the pear genome remains unclear.

### Unbiased distribution of LTR-RTs along the Pear Chromosomes

**Figure 2:**
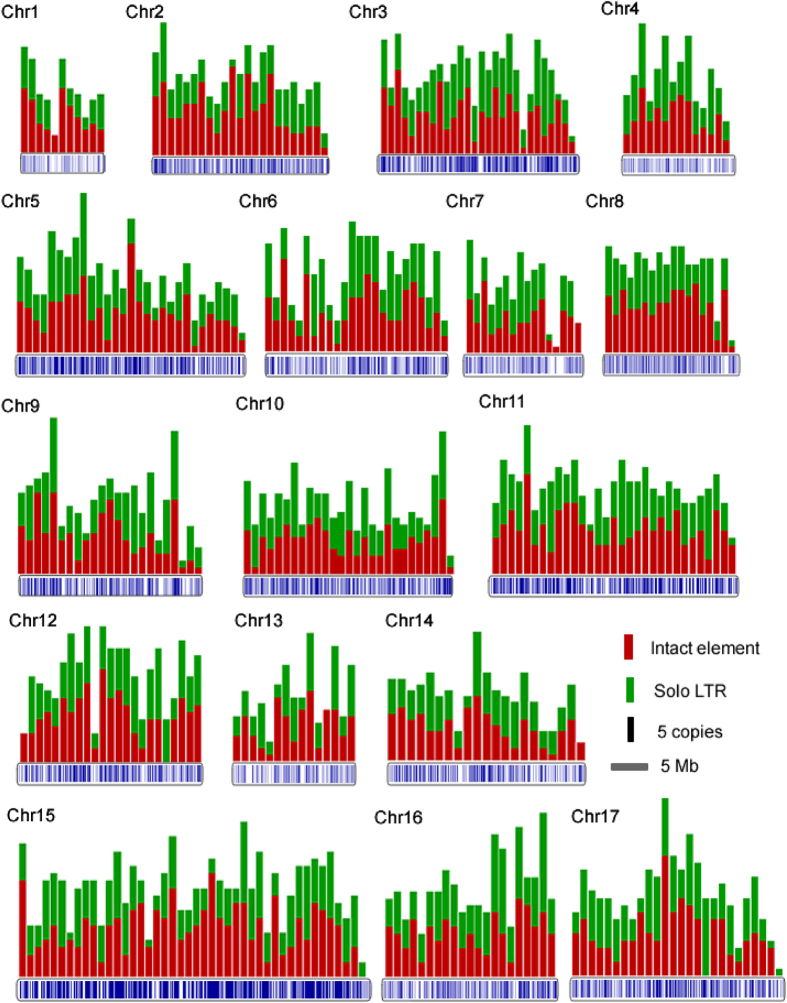
Distribution of LTR-RT copies through 17 pear (*P. bretschneideri)* chromosomes. Pear chromosomes and LTR-RT insertions are represented by grey horizontal boxes with blue vertical lines. Histograms over the horizontal boxes indicate the copy number of LTR-RT copies per Mb.

Generally plant chromosomes can be separated into gene poor heterochromatins (recombination-suppressed pericentromeric regions) and gene rich euchromatins (chromosome arms). Although several LTR-RT families have shown a bias towards integrating into or close to genes^27^,^32^, most of the LTR-RTs are usually found in the gene-poor heterochromatins^10^,^33^,^34^,^35^. In order to understand the distribution pattern of LTR-RTs in the pear genome, we initially made a randomization test for the genomic distribution of LTR-RT elements (IT and ST) according to the method previously described for the *PbrCassandra*^27^. Interestingly, unlike the distributions observed in other plant genomes, over 90% of the 6117 LTR-RT elements (IT and ST) are randomly distributed in the pear genome (Fig. 2, Table S1). To avoid the effect of *PbrCassandra* elements, which have shown an overall unbiased distribution along the pear genome^27^, we have made another randomization test only using the 4836 newly identified elements in this study. The new data showed a pattern consistent with all the IT and ST LTR-RTs (Figure S1). The LTR-RTs in 344 1-Mb windows (91%) show no statistical difference with those from a computational simulation (Figure S1). To eliminate the impact of window size on the randomization test, the 50-Kb and 100-Kb window distributions have also been tested, the LTR-RTs in 3786 100-Kb windows (98.90%) (Figure S2) and 7581 50-Kb windows (99.16%) (Figure S3) also show no statistical difference, indicating that most pear LTR-RTs may randomly distributed along the pear genomes. It should be pointed out that this unbiased distribution is the consequence of both insertion and elimination of LTR-RT DNA. Because the abundance and distribution of LTR-RTs may also be influenced by the quality of sequence assembly^36^, the unbiased insertions of LTR-RTs could also be caused by an issue with incorrect assembly. To answer this question, first, the non-assembled raw reads were used to estimate the abundance of the *Copia* and *Gypsy*-like elements through Bowtie software with default parameters. Comparing with the proportion of 16.9% and 25.5% in the assemble sequences of Oriental pear, there is no significant difference with the ratio of 20.21% and 27.85% in the raw reads (*P* = 0.1069). Second, we have analyzed the distributions of all the genes along the pear chromosomes as a control. The 34571 genes mapping to the assembled chromosomes were assigned into 378 non-overlapped 1-Mb windows. The randomization test showed that the genes in the 238 windows (~63%) were statistically different from those under a computational simulation (Figure S4), indicating that most of the genes in the pear may have a bias along the chromosomes. Taken together, the unbiased distributions of LTR-RTs may not be caused by incorrect assembly of the pear genome.

### Differential Spectrums of Activities from Different Groups of LTR Retrotransposons

**Figure 3:**
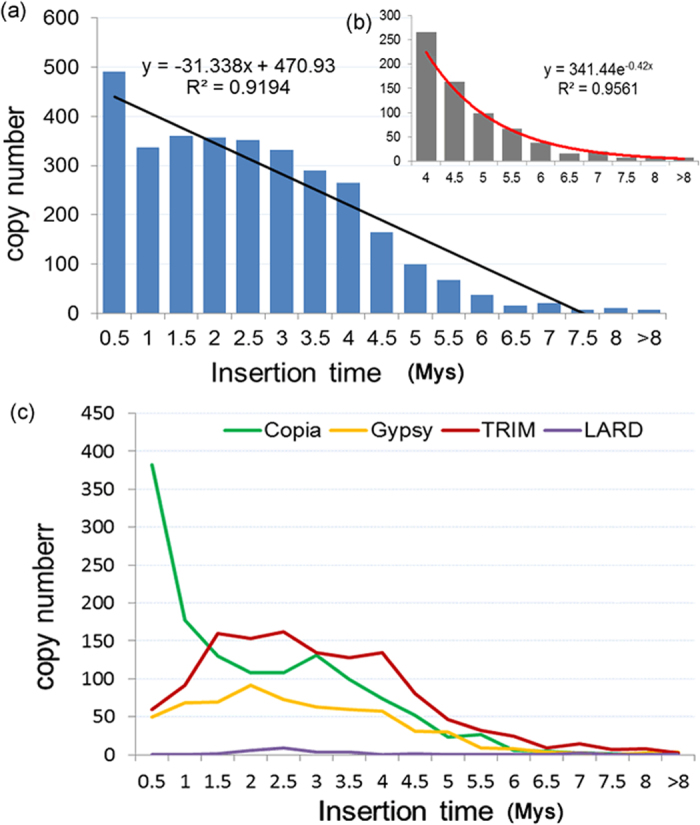
Insertion times of LTR-RT intact elements. (**a**) The insertion times distribution of 3221 LTR-RT with TSDs. (**b**) The insertion times distribution of 429 relatively old elements (over 4 Mys). (**c**) Comparison of insertion times of 1325 *Copi*a, 619 *Gypsy*, 1252 TRIM, and 24 LARD LTR-RT copies.

In order to understand the insertion time of LTR-RTs, the 3221 intact elements with TSDs have been aged using the approach previously described^37^,^38^. The data showed that 2782 (86.4%) elements proliferated in the last 4 Mys, and only 491 (15.3%) elements were generated in the last 0.5 Mys. A total of 121 (3.8%) elements were aged at 0 Mys, indicating that they may still be active (Fig. 3a, Table S1). It is interesting that, different from what has previously been described in soybean^10^, *Medicago*^11^ and rice^25^, the overall insertion times of all the intact elements in pear were not exponential but are negatively linearly correlated with the copy numbers (Fig. 3a, r = −0.96, *P* < 0.001, *Pearson* test). However, if only the relatively old elements (4 Mys) were calculated, the age distribution of LTR-RTs fits well with an exponential curve (Fig. 3b, r = 0.95 *P* < 0.001, *Pearson* test).

In order to further understand the distribution of LTR-RTs in pear, we investigated and compared the abundance, activities, and amplification timeframes of the LTR-RTs from different super-families. This effort yielded several clear observations 1) the average insertion time of LTR-RTs for *Copia*-like, *Gypsy*-like, TRIM and LARD super-families is 1.36, 2.22, 1.75, and 3.17 Mys, respectively; 2) most of the *Copia*-like elements (559, 42.2%) are amplified in the last 1 Mys, whereas most *Gypsy*-like (481, 77.7%), TRIM (962, 76.8%), and LARD (21, 87.5%) elements proliferated during the last 1–4 Mys; 3) Of the 491 LTR-RTs that proliferated within the last 0.5 Mys, 382 (77.8%) elements belong to *Copia*-like super-family; 4) Of the 121 elements with two identical LTRs, 113, 8, 0 and 0 are *Copia*-like, *Gypsy*-like, and TRIM or LARD elements, respectively (Fig. 3c).

### *Env*-like protein of *Copia Maximus* Lineage may be Lost During the six Rosaceae Species Evolution

The evolutionary relationships of individual LTR-RT families have been studied in several plant species^10^,^11^,^39^,^40^. For example, 88 *Copia*-like families, including 46 families from rice, 20 families from barley and wheat, and 22 families from *Arabidopsis* can be separated into six major evolutionary lineages, such as *Angela, Ale, Bianca, Ivana, Maximus, and TAR*. While *Gypsy*-like elements from sugarcane have been grouped into seven major evolutionary clades, such as *Tekay, Galadriel, CRM, Reina, Athila, Ogre* and *Tat*^39^.

**Figure 4:**
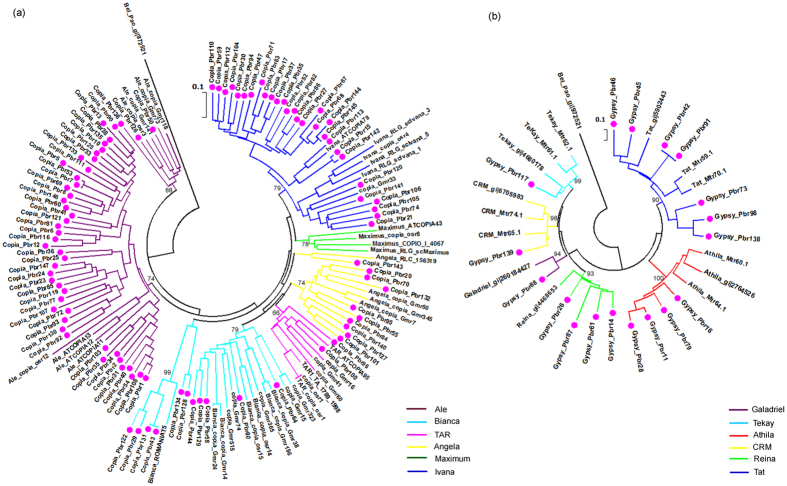
RT phylogenetic relationship of 117 LTR families identified in pear (*P. bretschneideri*). (**a**) 99 *Copia* families. (**b**) 18 *Gypsy* families. In each tree, a *Bel-Pao* type RT (gi#972521 from Genebank) of *Bomby xmori* is used as outgroup. Pink circles represent LTR-RT families from pear, and individual families are described by name and superfamily label. The lineage reference sequences described by lineage names are available in Repbase (Du, *et al.* 2010; Wang and Liu, 2008 and Wicker and Keller, 2007).

In order to understand the evolutionary history and phylogenetic relationships of individual families in pear, we performed phylogenetic analysis using the consensus DNA sequences from conserved RT domains in pear. As a result, 99 *Copia-*like families in pear have been grouped into five distinct lineages, *Ivana*, *Ale*, *Angela*, *TAR*, and *Bianca* (Fig. 4a), and the 18 *Gypsy-*like families in pear have been separated into six distinct evolutionary lineages, *Tekay, Galadriel, CRM, Reina, Athila,* and *Tat* (Fig. 4b). Interestingly, five out of the six *Copia-*like lineages are shared by pear and other plant species, but the *Maximus* lineage was not found in the pear genome (Fig. 4a), which may be caused by the likely accumulated mutations including the deletion of the RT protein sequence and became non-autonomous and truncated elements. Previous studies have indicated that *Maximus* is the only *Copia-*like lineage that contains the third ORF in the region between *Pol* and 3′LTR, encoding a hypothetical protein similar to *env*-like protein^10^,^41^,^42^,^43^. The *Maximus* lineage with *env*-like protain has been found in many Monocot species, such as rice and sugarcane^39^, as well as several eudicot species, including soybean^10^, *Medicago*^11^, and *Arabidopsis*^40^. Therefore, we investigated whether the *env*-like protein of *Maximus* lineage is also absent in other closely related genomes. To answer this question, we performed tblastn searches against the two pear genomes and seven other phylogenetically closely related genomes using the putative *env*-like protein sequences as queries. The data showed that the *Maximus env*-like protein is present in the rice (*Oryza sativa*)^29^, cucumber (*Cucumis sativus*)^44^, mulberry (*Morus notabilis*)^45^, and Cannabis (*Cannabis sativa*)^46^ genomes, but has been lost in all the other six Rosaceae genomes (no tblastn hit), including woodland strawberry (*Fragria vesca*)^47^, mei (*Prunus mume*)^48^, peach (*Prumus persica*)^2^, apple (*Malus domestica*)^49^ Oriental pear (*Pyrus bretschneideri*)^22^ and Occidental pear (*Pyrus communis*)^23^ (Figure S5). Since both monocot and eudicot species contain this *env*-like protein, it is possible that the *env*-like protein of *Copia Maximus* lineage may have been lost in the six Rosaceae species during the divergence of Rosales plants about 88.2 Mya^45^. However, because the assembled six Rosaceae genome sequences were generated by the whole genome shotgun (WGS) approach, and unavoidably contain many sequence gaps, thus whether the *env*-like protein of *Copia Maximus* lineage was truly lost in the six or even other un-sequenced Rosaceae species still need to be testified by experiments or more highly improved assembled genome sequences in the future.

The numbers of families and elements within each lineage can reflect the scales and timeframes of activity for proliferation of LTR-RTs among lineages and species^10^. To understand the amplification of individual families, we have calculated the copies and families in each lineage. *Bianca* is the *Copia-*like lineage with the highest copies (714, 44.3%), and these elements belong to 11 families, accounting for 11.1% of the 99 *Copia* families analyzed. In contrast, the *Ale* lineage contains the largest number of families (47, 47.5%), but has relatively fewer elements (350, 21.7%). In the six *Gypsy-*like lineages, *Tat* owns not only the largest number of LTR-RT families (7, 38.9%), but also the highest copies (1106, 61.5%). The *Galadriel, CRM,* and *Tekay* lineages each contain only one family, and the copies are 7, 8, and 214, respectively (Table 2, Table S2). *PbrCassadra/Pbr148* is the family with the highest number of copies in pear, belonging to TRIM group, and accounts for 33.3% of all the LTR-RTs identified in pear (Table S2). However, it is difficult to classify it into *Copia* or *Gypsy* superfamilies due to the lack of any genes related with transposition.

### Orthologous LTR-RTs and Single Copy Genes Reveal Similar Evolutionary Rates between *P. bretschneideri* and *P. communis*

The recent release of the *P. communis* draft genome sequence allows a comparative analysis of nucleotide divergence between the two *Pyrus* species. To do this, we first identified the orthologous LTR-RT elements between the two genomes using a previously described method^27^,^35^,^37^ (Figure S6). This method was based on the unique sequence of each TE junction site, and the orthologous insertion was defined if the junction sequence only has one best match in the genome. Under these criteria, a total of 1194 elements (19.5%), including 656 intact elements (20.4%) and 538 solo LTRs (18.6%) with TSDs were found in the draft assembled *P. communis* genome (Table S1). Due to the assembly issue, most of these shared elements were truncated, and only 33 orthologous intact elements with TSDs were identified in the *P. communis* genome (Table S1). To further verify the orthologous relationships of LTR-RTs, 5 out of 33 shared LTR-RT insertions were randomly detected using the PCR method (see Methods and Materials). The observed junction size of each insertion was consistent with the estimate based on the bioinformatics approach (Figure S7, Table S3), indicating that the identified orthologous LTR-RTs are indeed shared by the two pear genomes. However, because the coverage of NGS reads and assembly quality from the Occidental pear genomes used in the orthologous LTR-RTs analysis remain low (11.4 X genome coverage and 8.8 Kb N50 size), the proportions of orthologous LTR-RTs between the two *Pyrus* genomes were likely to be underestimates.

**Figure 5:**
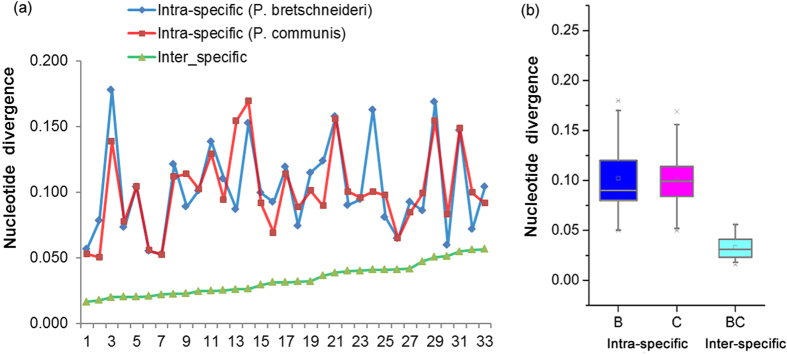
Intra- and inter-specific sequence divergence evaluated based on orthologous LTR-RTs shared by the *P. bretschneideri* and *P. communis* genomes. (**a**) Intra-specific sequence divergence between two LTRs of each of the 33 LTR-RTs shared by *P. bretschneideri* and *P. communis*, and inter-specific sequence divergence between the two genomes at these 33 LTR-RT sites. (**b**) Boxplot of intra-specific and inter-specific sequence divergence between two LTRs of each of the 33 orthologous intact LTR-RT shared by *P. bretschneideri* and *P. communis*. The bottom and top boundaries of the box are the first and third quartiles, and the bold lines within individual boxes are the medians, referred to as the second quartiles, the short bold lines within individual boxes indicate the mean values of the data. The ends of the whiskers (the dotted lines) represent minimum and maximum values.

Theoretically, the genomic sequences of the two *Pyrus* species should be identical at the time when they split from a common ancestor, and the two orthologous copies of LTR-RTs have evolved independently since then. Therefore, the evolutionary rates between and within the two genomes can be estimated by comparing the nucleotide divergence between the orthologous elements. The data from the 33 orthologous intact LTR-RTs showed that intra-specific sequence divergence of the two LTRs within each individual element is significantly higher than the inter-specific sequence divergence (*P* < 0.01, *t-*test) (Fig. 5a). There was no significant difference observed between the intra-specific comparisons for the divergence of two LTRs (*P* = 0.5974, *t-*test) (Fig. 5a,b, Table S1 and Table 3). These data also indicate that the 33 orthologous elements might have been inserted into the genome before the split of the two species, and that orthologous LTR-RTs evolved at similar rates after the split.

To compare the evolutionary rates of orthologous LTRs with the genes, we investigated the divergence of genic sequences. Here, we have identified 774 high confidence orthologous single genes in total between the two *Pyrus* genomes (Table S4) according to previously described method^22^ (also see Materials and Methods). In order to shed light on the divergence pattern of these orthologous single genes, we selected *A. thaliana, M. domestica and P. persica* as a reference genome, respectively. A total of 299, 293 and 303 high confidence orthologs (out of 774) were identified in *A. thaliana, M. domestica and P. persica* through the same method (see Materials and Methods). Then we aligned each of the 299, 293 and 303 single genes in the two genomes with their putative orthologs in the three reference genomes and were separately able to calculate Ka, Ks, and ω for each of 299, 293 and 303 orthologous single genes in *P. bretschneideri* and *P. communis* versus their respective orthologs in *A. thaliana* (Table S5), *M. domestica* (Table S6) *and P. persica* (Table S7), respectively. There is no significant difference of Ks and Ka (*P* > 0.05) between the two pear genomes (Table 3), indicating that similar evolutionary rates were observed not only in LTR-RTs but also in genic sequences.

### Comparisons of Nucleotide Substitution Rates between LTR-RTs and genes, and Estimation of the Divergence Time between *P. bretschneideri* and *P. communis*

**Figure 6:**
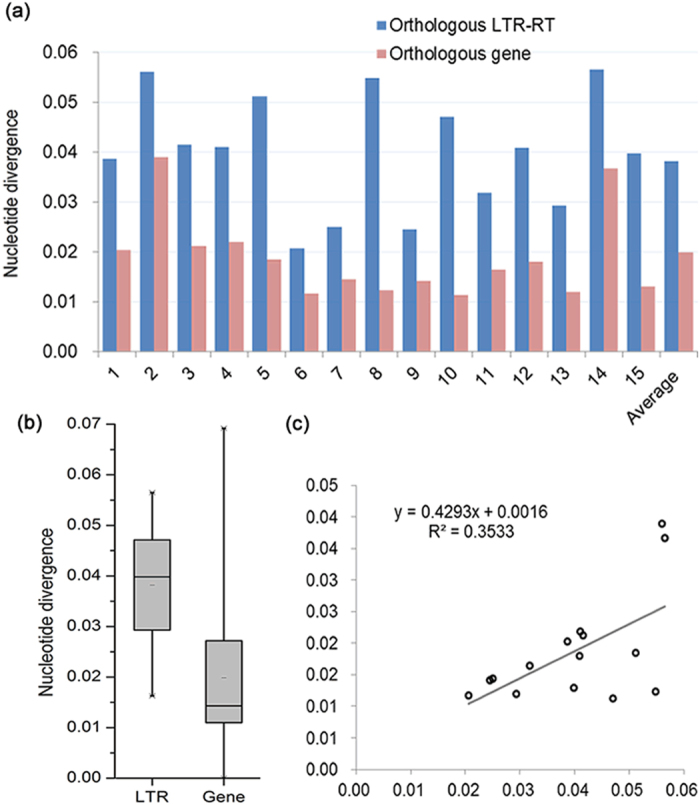
Comparisons of inter-specific sequence divergences of orthologous LTR-RTs and single genes retained in 15 1-Mb syntenic regions of *P. bretschneideri* and *P. communis* genomes. (**a**) Histogram comparisons of inter-specific sequence divergences of orthologous LTR-RTs and single genes. *x* axis represents 15 1-Mb syntenic regions of *P. bretschneideri* and *P. communis* genomes, named by the orthologous LTR-RTs. (**b**) Boxplot comparisons of inter-specific sequence divergences of orthologous LTR-RTs and single genes. (**c**) Correlation between inter-specific sequence divergences of orthologous LTR-RTs and single genes. Data was shown in Table S8.

Nucleotide substitution rates vary significantly in different genes, genomic sites, and lineages^50^,^51^. For example, LTR-RTs have been found to diverge more rapidly than genes^52^,^53^, and a later study revealed that nucleotide substitution rates in LTR-RTs were almost two-fold higher than of genic sequences between two rice subspecies^37^. In another study, however, the substitution rates of LTR-RTs were found to be even five to six-fold higher than in genic regions between two rice subspecies^54^. To compare the evolutionary rates between LTR-RTs and genes in pear, we have investigated 33 1-Mb orthologous regions (0.5-Mb upstream and 0.5-Mb downstream) between the two *Pyrus* species containing one orthologous LTR element (Table S8). As shown in Fig. 6a,b, the divergence (measured as K) of orthologous LTR-RTs (0.0382 ± 0.0123) is significantly higher than the Ks of orthologous genes (0.0199 ± 0.0136) (*P* < 0.01, *t-*test), about two-fold higher in the former. The data also showed that inter-specific divergence of two LTR sequences in one element is positively correlated with Ks (*r* = 0.594, *P* < 0.01, *Pearson* test) (Fig. 6c), indicating that the divergence of orthologous LTR-RTs can also reflect the evolutionary rate in a genome.

**Figure 7:**
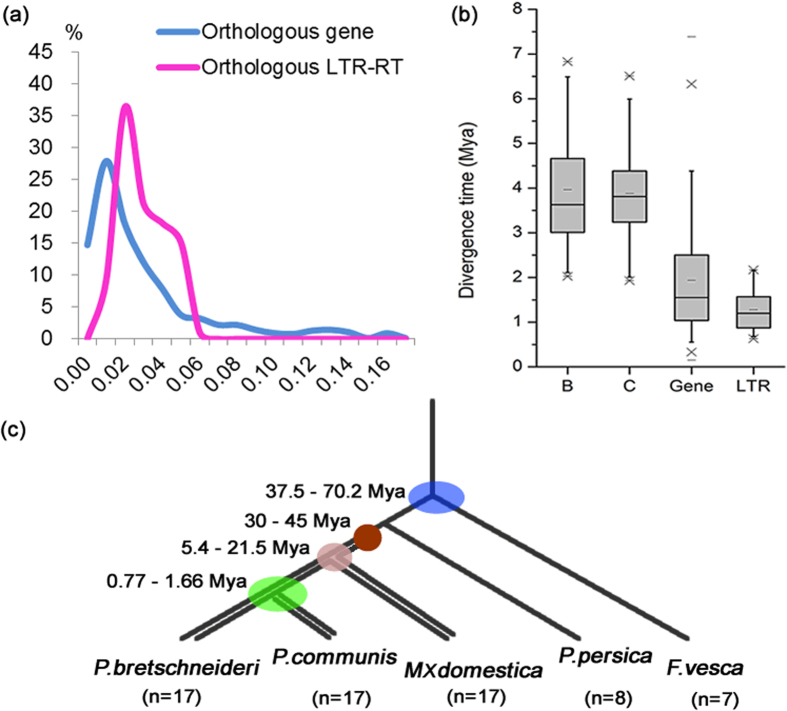
Divergence time between *P. bretschneideri and P. communis.* (**a**) Ks and K distributions of orthologous gene and LTR-RT in *P. bretschneideri and P. communis.* Ks indicates substitutions per synonymous site; K represent the nucleotide divergence of orthologous LTR-RT. (**b**) Boxplot comparison of orthologous LTR-RT insertion times and estimated divergence time between *P. bretschneideri and P. communis. ‘*B’ and ‘C’ represent insertion times of orthologous LTR-RTs from *P. bretschneideri and P. communis, respectively. ‘*Gene’ and ‘LTR’ refers to the divergence time between *P. bretschneideri and P. communis* estimated by the Ks and K of orthologous gene and LTR-RT, respectively. (**c**) Phylogenetic relationships and divergence time between *F. vesca, P. persica, M x domestica, P. bretschneideri and P. communis*. Mya, million years ago.

We have also investigated the distribution of evolutionary rates in LTR-RTs and genes. As shown in Fig. 7a, both Ks (between orthologous genes) and K (orthologous LTR-RTs) distribution showed only one peak. About 27.91% of the Ks ranges between 0.01 and 0.02, and 36.36% of the K were between 0.02 and 0.03 (Fig. 7a), suggesting that LTR-RTs evolved much faster (~1.5–2 times) than genes. The peaks of Ks and K might represent the divergent event between *P. bretschneideri* and *P. communis*^22^. Using an evolutionary rate of 6.03 × 10^−9^ substitutions per site per year for *Adh* gene^55^, the split time between the two *Pyrus* species was estimated to have occurred at 0.83–1.66 Mya. Using the evolutionary rate 1.3 × 10^−8^ per site per year for LTR-RTs^56^, we estimated that the divergence event occurred at 0.77–1.15 Mya. Therefore we speculate that the divergence time between the two *Pyrus* species might have been 0.77–1.66 Mya (Fig. 7b,c).

## Discussion

### Unbiased Distribution as a Unique Feature of LTR Retrotransposons in the Pear Genome

One of the most interesting findings of this study is the observation that most LTR retrotransposons are randomly distributed in the pear genome. Although our previous work on *Cassandra* retrotransposons showed similar distribution in the pear genome, unbiased locations of plant LTR retrotransposons observed in the whole genome level has not yet been reported. For example, at least 87% of soybean LTR retrotransposons were found in recombination-suppressed pericentromeric regions^10^. In the rice genome with a smaller genome size, the densities of LTR retrotransposons in the pericentromeric regions is >1.5 fold higher than in chromosome arms^25^. Our recent study on tomato plants also indicates that the LTR retrotransposon density in gene-poor heterochromatic regions (23.1 per Mb) is greater than in euchromatic regions (7.93 per Mb), indicating that different chromatin structure may be a determinate factor of LTR retrotransposon density^36^. Even in the much more compact *Arabidopsis* genome, non-random genomic distribution was observed and explained by both selection against insertion in euchromatin and preferential targeting of heterochromatin^57^. Therefore, the overall unbiased distribution may represent a unique feature of LTR retrotransposons in the pear genome.

The causes and factors that result in the distribution of pear LTR retrotransposons remain mysterious. First, unlike the rice genome, where genomic components are organized according to the local genomic rates^25^, neither the number of LTR retrotransposons nor the number of genes is correlated with genetic recombination rates in pear (Figure S8, and S9), indicating that the distribution profiles of genomic DNA in pear are less affected by recombination rate. Second, insertion bias is another factor that could affect the distribution of LTR retrotransposons^57^. In the pear genome, >98% of relatively young LTR retrotransposons (insertion time <1Mys, representing the status of initial integrating) (Figure S1) are randomly dispersed in the genome, suggesting that unbiased integrating of LTR retrotransposons may indeed occur in the pear genome. It should be noted that the distribution pattern of LTR-RTs is the balance of both insertion and selection, and thus selection intensities in different genomic region also contribute to the LTR-RT densities. Third, many plant genomes harbor a large proportion of recombination suppressed heterochromatin. For instance, >10% of the rice^25^, >50% of the soybean^10^, and >70% of the tomato^36^ genomic DNA are composed of gene-poor heterochromatin. In contrast, in the 17 pear chromosomes, no or very little heterochromatin can be identified by comparing genetic and physical maps (Figure S8). Moreover, 943 out of the 1334 unanchored scaffolds (70.69%) were detected harboring LTR-RTs and related fragments, and the size of scaffolds containing LTR-RTs are not only positively correlated with the insertion number of LTR-RTs (Figure S10a, r = 0.512, *P* < 0.01, *Pearson* test) but also significantly bigger than those scaffolds without LTR-RTs (Figure S10b, *P* < 0.01, *t*-test), indicate that the unmapped LTR-RTs were also widely distributed in the unanchored scaffolds with largely affected by the scaffold size. Furthermore, the ratio of left unmapped LTR elements (23.7%) is almost the same with the proportion of unanchored DNA sequences, and the 5,532 (76.3%) LTR elements mapped on the 75.5% anchored 17 chromosomes can also reflect that the less heterchromotin maybe not caused by poor assemble of pericentromeric region. Therefore, it is reasonable to hypothesize that the lack of “pericentromeric effects” could be one of the causes shaping the distribution of LTR retrotransposons in pear. In summary, the lack of correlation with genomic rates, unbiased integrating, and little “pericentromeric effects” are probably three factors that are responsible for the overall unbiased distribution of LTR retrotransposons in the pear genome.

### Differential Spectrums Activities of LTR Retrotransposons in the Pear Genome

Although individual LTR retrotransposon may have different timeframes, comprehensive analysis of several plant genomes has shown that most intact elements were dated to <1Mya, and the overall age distribution fits an exponential decay^10^,^11^,^40^,^57^. In the pear genome, however, the insertion time of intact elements does not fit an exponential distribution, but exhibits a negative linear correlation with the copy numbers (Fig. 3a). This could be partially explained by differential activities of different groups of intact elements. Based on the active timeframes of LTR retrotransposons, the evolution of the pear genome can be artificially separated into three stages. During the period >4 Mya, DNA loss of retrotransposon is exponentially correlated with age, resulting in an overall exponential curve between the copy numbers of intact elements and the insertion time (Fig. 3b). In the period 1–4 Mys, however, the *Copia*, *Gypsy*, and TRIM elements have continuous high activities, accumulating a large number of copies (Fig. 3c). In the recent 1 Mys, *Copia* elements were dramatically amplified, whereas *Gypsy* and TRIM elements have only weak activity (Fig. 3c). Differential amplification of LTR retrotransposons has also been detected in different genomic regions, such as euchromatins and heterochromatins. Our recent work has indicated that the highly suppressed activity of intact elements in gene-poor heterochromatins could be a major reason for the biased distribution of young elements in tomato plants^36^. These data suggest that each genome may have its unique characteristics and evolutionary history, which could influence the overall age distribution of LTR retrotransposons.

### Similar Evolutionary Rates of LTR Retrotransposons in Different Pear Genomes

A recent comparative genomic analysis of two Brassica species, B. *rapa* and B. *oleracea* (which split from their common ancestor ~3.75 Mya), has revealed that the nucleotide evolutionary rate in the former is much higher than in the latter^58^. This asymmetric evolution of two genomes from the split of their common ancestor has been explained by different genetic recombination^58^. This is a reasonable deduction since an association between nucleotide divergence and genetic rate has been observed^58^, and recombination is assumed to facilitate the generation of point mutations^59^. In this study, however, similar evolutionary rates of LTR retrotransposons have been detected in the two *Pyrus* species. One simple explanation may be that the two pear genomes share similar genetic rates. Although the occidental pear genome is only poorly assembled^23^, the recent split time between the two pear genomes estimated from this study (0.77~1.66 Mya), has indicated that their genomic features may not change much. Further investigation and detailed analysis of the two genomic sequences may be valuable for the understanding of their DNA components, genomic features, evolutionary history, and the better utilization for pear breeding in the future.

## Experimental Procedures

### Genome sequence resources and annotation of LTR-RTs

The assembled oriental pear (*P. bretschneideri*) genome sequence (Pbr_V1.0), predicted CDS and protein data sets are available at the Pear genome project website (http://peargenome.njau.edu.cn/) and *GigaDB* website (http://gigadb.org/site/index). Meanwhile, the assembled occidental pear (*P. communis*) genome sequence, together with the annotated CDS and protein data sets were downloaded from the Phytozome website (http://www.phytozome.net).

A combined strategy based on the structural analysis and sequence homology comparisons was employed to identify the LTR-RT elements in the 17 assembled pear (*P. bretschneideri*) chromosomes. Initially, intact elements were identified by LTR_STRUC program^60^. Then the LTR sequences of the intact elements with clearly defined boundaries were used to detect additional intact elements (without TSDs) and solo LTRs (with or without TSDs) by sequence homology searches using CROSS_MATCH and CLUSTALW program with default parameters, and the TSDs sites were defined with one mismatch allowed^10^,^37^. The structures and boundaries of all of the identified LTR-RTs were confirmed by manual inspection, fragments and truncated elements were not analyzed in this study. The LTR-RTs were classified into *Copia*-like and *Gypsy*-like, TRIM and LARD superfamilies, and individual families by sequence homology comparison, which were defined by the criteria described previously^6^,^11^.

### The distribution of LTR-RTs and genes

According to previously described method^25^,^27^, each assembled pear chromosome was split into contiguous 1-Mb windows, and the last window (<1-Mb) for each chromosome was not included in this analysis. GR rates were plotted on the basis of midpoints of each window. Only intact LTR-RTs and solo LTRs flanking with TSDs were selected for the distribution densities analysis. The distributions and densities of genes were obtained from the latest annotation of Pbr_V1.0 chromosomes (http://gigadb.org/site/index) with modifications. Genes matching TEs and hypothetical genes were excluded. An LTR-RT or gene was assigned to a particular window based on its midpoint. The windows with >0.5 Mb “N” were not included in the correlation analysis. “N”s, if any, in the 1-Mb contiguous windows were not counted.

Randomization analysis followed a previously described method^27^. The correlations of GR rates with LTR-RT densities and gene densities were assessed using *Pearson’s* correlation by 10,000 bootstrap re-samplings implemented in the SPSS software.

### Estimation of GR rates

The local GR rates were estimated by using MareyMap^61^. A total of 2005 markers selected from the genetic map of pear^62^ was anchored to the genomic sequence of the pear genome (Pbr_V1.0 chromosomes), on the basis of their best matches (>95% in identity and >95% in length) and consistent orders in physical and genetic maps.

### Dating of insertion time and divergence time

Since the two LTRs of an element are identical at the time of insertion, the insertion time of an element can be roughly dated based on the sequence divergence of two LTRs by employing an appropriate mutation rate^38^. For the LTR-RT elements shared by two closely related species, the nucleotide divergence of two orthologous LTRs can be calculated to estimate the divergence time between the two genomes. This approach has been used in tomato^63^, rice^54^, and two *Brassica* species, *B. rapa* and *B. oleracea*^58^.

The insertion time of each intact LTR–RT and divergence time of orthologous LTR-RTs were aged by a previously described method^10^,^63^. An average substitution rate (r) of 1.3 × 10^−8^ substitutions per synonymous site per year and the insertion time (T) formula T = k/2r were employed to convert sequence divergence into insertion time and divergence time^37^.

### Phylogenetic and *Maximus* lineage analysis

Typical *Copia*-like or *Gypsy*-like conserved RT cDNA sequences were extracted from the intact consensus sequences of individual families. Sequence alignments were performed by MUSCLE3.8.31 program with default options^64^. MEGA 5.0 program implemented with *Jukes–Cantor* model was employed for the neighbor-joining tree building^65^. Twelve putative *env*-like protein sequences from the National Center for Biotechnology Information website (GeneBank: AAO73528.1, AAO73526.1, AAO73524.1, AAO73522.1, AAO73530.1, AAC64918, AAG52950.1, AAO73528.1, AAO73526.1, AAO73524.1, AAO73522.1, and AAO73530.1) have been used as queries to perform tblastn searches (Evalue = 1e−5) against seven other phylogenetically closely related genomes.

### Identification of orthologous LTR-RTs between the two pear genomes

A strategy based on the previous studies was implemented to identify the insertions of orthologous LTR-RT copies between the two pear genomes (Figure S6)^35^,^56^,^58^. Only intact or solo LTR-RT elements flanking with TSDs and with unique junction sites from *P. bretschneideri* were selected for BLASTN searches against the *P. communis* genome. Two 100-bp (50-bp flanking sequences and 50-bp LTR-RT terminal sequence) junction sequences were extracted as query databases for BLASTN searches against the *P. communis* genome sequences. In this approach, an element was considered to be orthologous between the two genomes when the 100bp junction sequences found were unique in the draft sequences of the *P. communis* genome.

### PCR analysis of orthologous LTR-RTs

Total genomic DNA of the pear cultivars ‘Dangshansuli’ (*P. bretschneideri* Rehd.) and ‘Bartlett’ (*P. communis* L.) were extracted from young leaves using the improved CTAB method. Five orthologous LTR-RT copies were randomly selected and their 600-bp junction sequences, including 300-bp 5′ flanking sequences and 300-bp 5′ LTR terminal sequences, were extracted and used to design primers, respectively (Table S3). PCR reactions were carried out in a 25 μL volume containing 1 μl of 50 ng/μl genomic DNA template, 2.5 μl of 10×buffer (without MgCl_2_), 2.5 μl of 2.5 mM dNTP mixture, 2.5 μl of 25 mM MgCl_2_, 0.8 μl each of forward and reverse primer (10 pmol/μl), and 0.2 μl of 5U/μl Taq polymerase (Takara Biotechnology Company, Dalian). The reactions were performed with the following conditions: 94 °C for 3 min, then 35 cycles of 94 °C for 30 s, 55 °C for 40 s, and 72 °C for 2 min, and a final step at 72 °C for 10 min. The products were resolved on 1% agarose and detected by EB (Ethidium bromide) staining. The analyses were performed three times and loaded on independent gels.

### Identification of single-copy orthologous genes and estimation of sequence divergence

A strategy has been developed for identification of single-copy orthologous genes between the two pear genomes based on a previous study^22^. First, the protein sequences of *P. bretschneideri* and *P. communis* were set as a database that was used to perform all against all BLASTP comparison with an e-value cut-off of 1e-05. On the resulting similarity matrix, orthoMCL software^66^ was used to perform a Markov clustering algorithm to define the gene cluster structure with a default MCL inflation parameter of 1.5. All the identified single-copy orthologous genes were manually inspected, and gene sequences that contained frame shift mutations or stop codons were excluded from further analysis. Single-copy orthologous genes between *P. bretschneideri* and each of the three reference genomes (*A. thaliana, M. domestica, and P. persica*) as well as between *P. communis* and each of the three reference genomes (*A. thaliana, M. domestica, and P. persica*) were also identified using the same strategy, respectively.

The Ka, Ks, and ω (Ka/Ks) of single-copy orthologous genes were calculated using the YN00 program in the PAML software package^67^. In addition, the Ka, Ks, and ω of the orthologous genes between *P. bretschneideri* and *A. thaliana, M. domestica, and P. persica*, and between *P. communis* and *A. thaliana, M. domestica, and P. persica* were compared using Student’s paired *t*-test.

## Additional Information

**How to cite this article**: Yin, H. *et al.* Genome-wide Annotation and Comparative Analysis of Long Terminal Repeat Retrotransposons between Pear Species of *P. bretschneideri* and *P. Communis*. *Sci. Rep.* **5**, 17644; doi: 10.1038/srep17644 (2015).

## Supplementary Material

Supplementary Information

Supplementary tables

## Figures and Tables

**Table 1:** Summary of LTR-RT superfamilies in pear.

Superfamily	Family		IT		ST		InT		SnT		Subtotal		Ratio	Ave. age
	No.	%	No.	%	No.	%	No.	%	No.	%	No.	%	(ST+SnT)/(IT+InT)	(mys)
*Copia*	115	77.7	1326	41.2	948	32.7	241	41.7	160	29.0	2675	36.9	0.71	1.36
*Gypsy*	21	14.2	619	19.2	1060	36.6	123	21.3	112	20.3	1914	26.4	1.58	2.22
TRIM	9	6.1	1252	38.9	846	29.2	212	36.7	270	48.9	2580	35.6	0.76	1.75
LARD	3	2.0	24	0.7	42	1.5	2	0.3	10	1.8	78	1.1	2.00	3.17
Total/Average	148	100	3221	100	2896	100	578	100	552	100	7247	100	0.91	2.13

IT, ST, InT, and SnT represent intact element with TSDs, solo LTR with TSDs, intact element without TSDs and solo LTR without TSDs.

**Table 2:** Summary of LTR-RT lineages in pear.

Superfamily Lineages	No. of family	No.of IT	No. of ST	No.of InT	No. of SnT	subtotal	Ratio (ST + SnT)/(IT + InT)	Ave. age (mys)
*Copia*								
*Ale*	47	296	33	14	7	350	0.13	1.03
*Ivana*	29	173	48	19	10	250	0.30	1.38
*Bianca*	11	455	116	70	73	714	0.36	1.51
*Angela*	10	49	117	43	14	223	1.42	1.27
*TAR*	2	12	55	2	4	73	4.21	1.87
Subtotal/average	99	985	369	148	108	1610	0.42	1.41
*Gypsy*								
*Athila*	4	76	318	22	33	449	3.58	1.99
*Tat*	7	433	531	80	62	1106	1.16	2.82
*Renia*	4	13	0	0	0	13	/	2.41
*CRM*	1	7	0	0	0	7	/	0.05
*Galadriel*	1	7	0	1	0	8	/	0.56
*Tekay*	1	20	175	7	12	214	6.93	1.37
Subtotal/average	18	556	1024	110	107	1797	1.70	1.53

IT, ST, InT, and SnT represent intact element with TSDs, solo LTR with TSDs, intact element without TSDs and solo LTR without TSDs.

**Table 3:** Inter-specific comparison of intra-element LTR sequence divergence and the evolutionary rates of orthologous singletons between *P. bretschneideri* and *P. communis*.

Genomic feature^a^	*P. bretschneideri*^b^	*P. communis*^b^	*P* value^c^
Nucleotide divergence between two LTRs of individual LTR-RTs	0.1030 ± 0.0343	0.1009 ± 0.0310	0.5974
Ka: compared with *A. thaliana*	0.3535 ± 0.1922	0.3598 ± 0.2163	0.0750
Ks: compared with *A. thaliana*	2.1578 ± 0.8774	2.1178 ± 0.8564	0.2690
ω (Ka/Ks): compared with *A. thaliana*	0.1806 ± 0.1015	0.1894 ± 0.1262	0.0412
Ka: compared with *M. domestica*	0.1129 ± 0.2455	0.1144 ± 0.2339	0.9066
Ks: compared with *M. domestica*	0.1810 ± 0.3579	0.1738 ± 0.3120	0.7121
ω (Ka/Ks): compared with *M. domestica*	0.5782 ± 0.3876	0.5838 ± 0.3794	0.6890
Ka: compared with *P. persica*	0.1452 ± 0.1777	0.1894 ± 0.1262	0.0504
Ks: compared with *P. persica*	0.4670 ± 0.4260	0.4686 ± 0.4190	0.8677
ω (Ka/Ks): compared with *P. persica*	0.3083 ± 0.1641	0.3136 ± 0.1694	0.1546

^a^Ka, Ks and ω (compared with *A. thaliana*, *M. domestica* and *P. persica*) of the 299, 290 and 303 genes (out of 774 single copy orthologous genes) were calculated based on their respective orthologos in *A. thaliana, M. domestica* and *P. persica*, respectively.

^b^Mean ± SD.

^c^Student’s paired *t* test.
