# Supplementary material for: Genome-wide Annotation and Comparative Analysis of Long Terminal Repeat Retrotransposons between Pear Species of P. bretschneideri and P. Communis
Source: Sci Rep. 2015 Dec 3;5:17644. doi: 10.1038/srep17644 (PMC4668562; doi:10.1038/srep17644)
Supplement: Supplementary Information [file srep17644-s1.doc]

**Genome-wide Annotation and Comparative Analysis of Long Terminal Repeat Retrotransposons between Pear Species of *P. bretschneideri* and *P. Communis***

Hao Yin^1^, Jianchang Du^2^, Jun Wu^1^, Shuwei Wei^1^, Yingxiu Xu^2^, Shutian Tao^1^, Juyou Wu^1^,

Shaoling Zhang^1*^

^1^Center of Pear Engineering Technology Research, State Key Laboratory of Crop Genetics and Germplasm Enhancement, Nanjing Agricultural University, Nanjing 210095, Jiangsu, China.

^2^Institute of Biotechnology, Jiangsu Academy of Agricultural Sciences, Nanjing 210014, Jiangsu, China.

*Corresponding author: Shaoling Zhang, Center of Pear Engineering Technology Research, State Key Laboratory of Crop Genetics and Germplasm Enhancement, Nanjing Agricultural University, Nanjing 210095, China; Tel:0086-25-84396580, Fax:0086-25-84396485, Email: slzhang@njau.edu.cn

Supplementary Figures

**
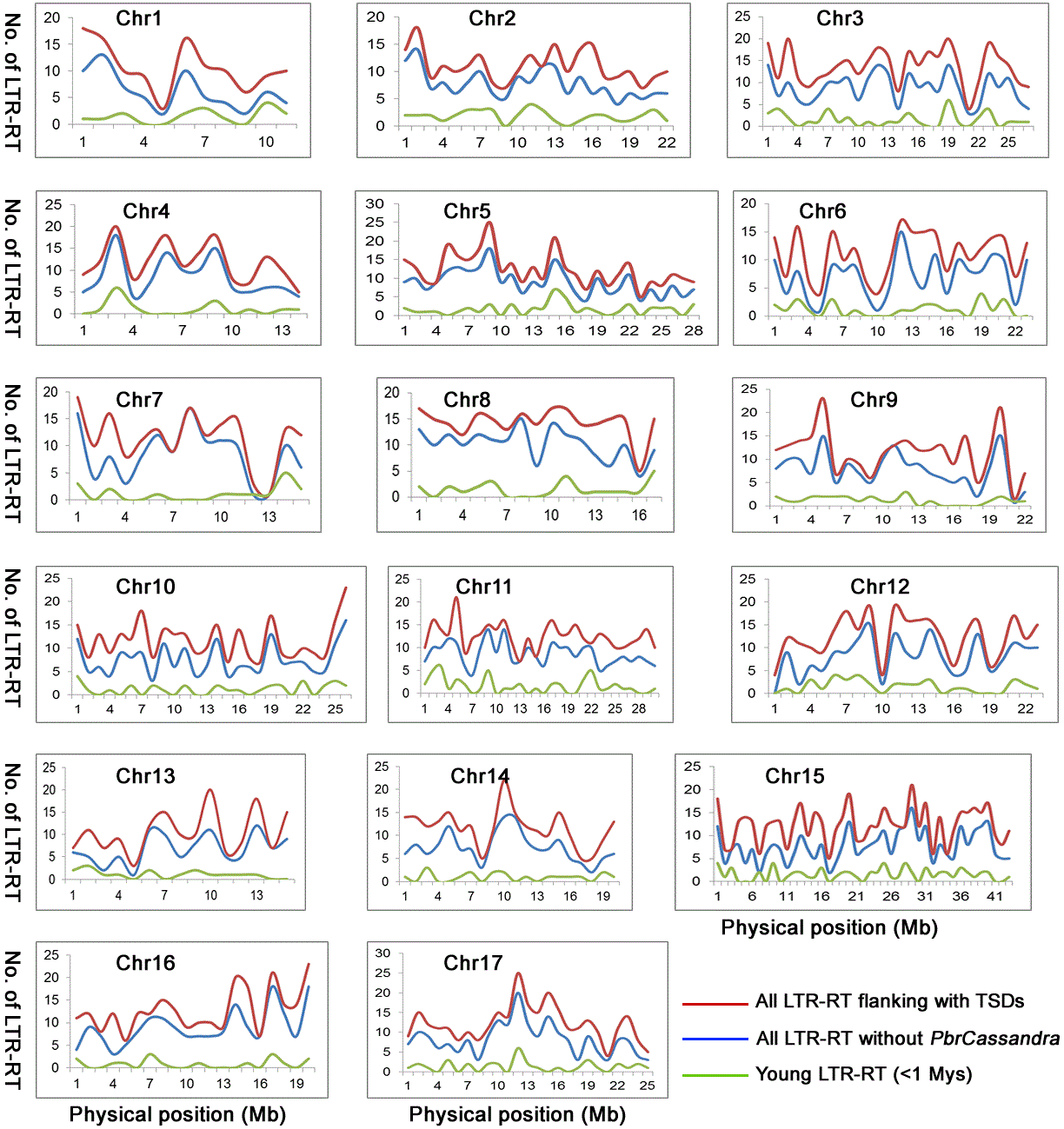
**

**Figure S1.** Distribution of anchored LTR-RTs through 17 pear (*P. bretschneideri*) chromosomes (1-Mb window).

Red lines indicate the density of all the intact LTR-RT and solo LTRs flanking with TSDs; Blue lines mean the density of intact LTR-RT and solo LTRs without *PbrCassandra* elements; Green lines refer to the density of young intact LTR-RTs aged miner than 1 mys.

**
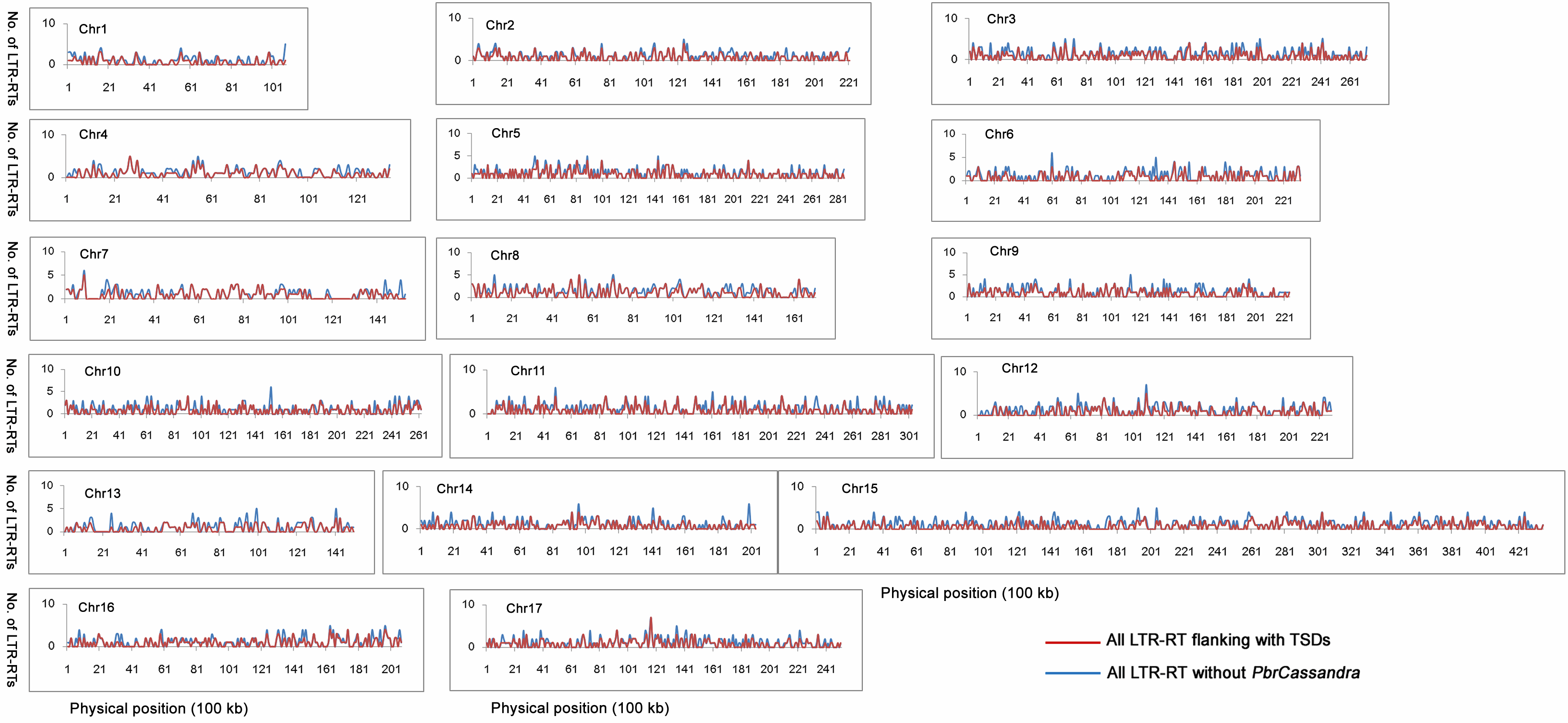
**

**Figure S2.** Distribution of anchored LTR-RTs through 17 pear (*P. bretschneideri*) chromosomes (100-Kb window).

Red lines indicate the density of all the intact LTR-RT and solo LTRs flanking with TSDs; Blue lines mean the density of intact LTR-RT and solo LTRs without *PbrCassandra* elements.

**
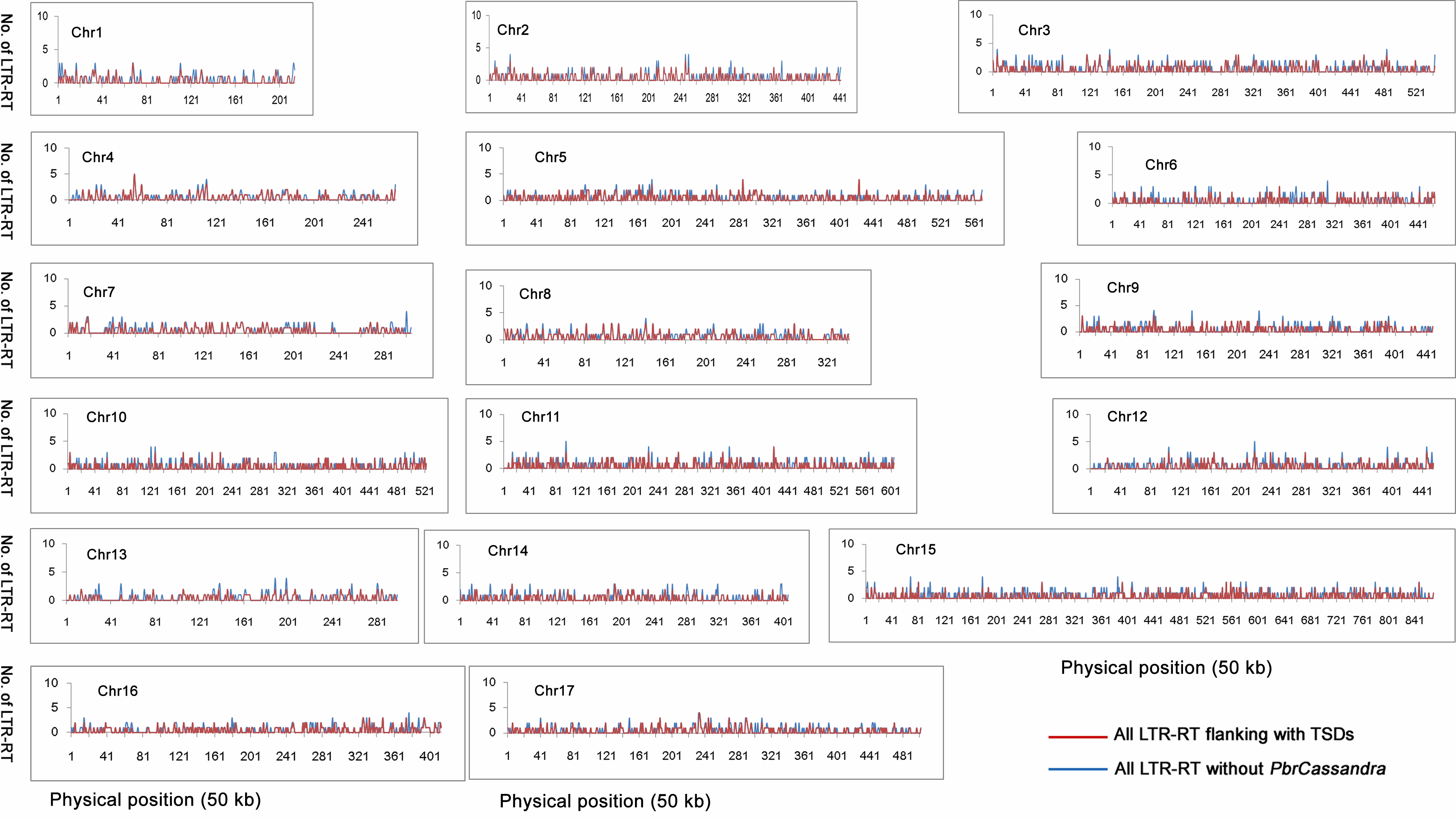
**

**Figure S3.** Distribution of anchored LTR-RTs through 17 pear (*P. bretschneideri*) chromosomes (50-Kb window).

Red lines indicate the density of all the intact LTR-RT and solo LTRs flanking with TSDs; Blue lines mean the density of intact LTR-RT and solo LTRs without *PbrCassandra* elements.

**
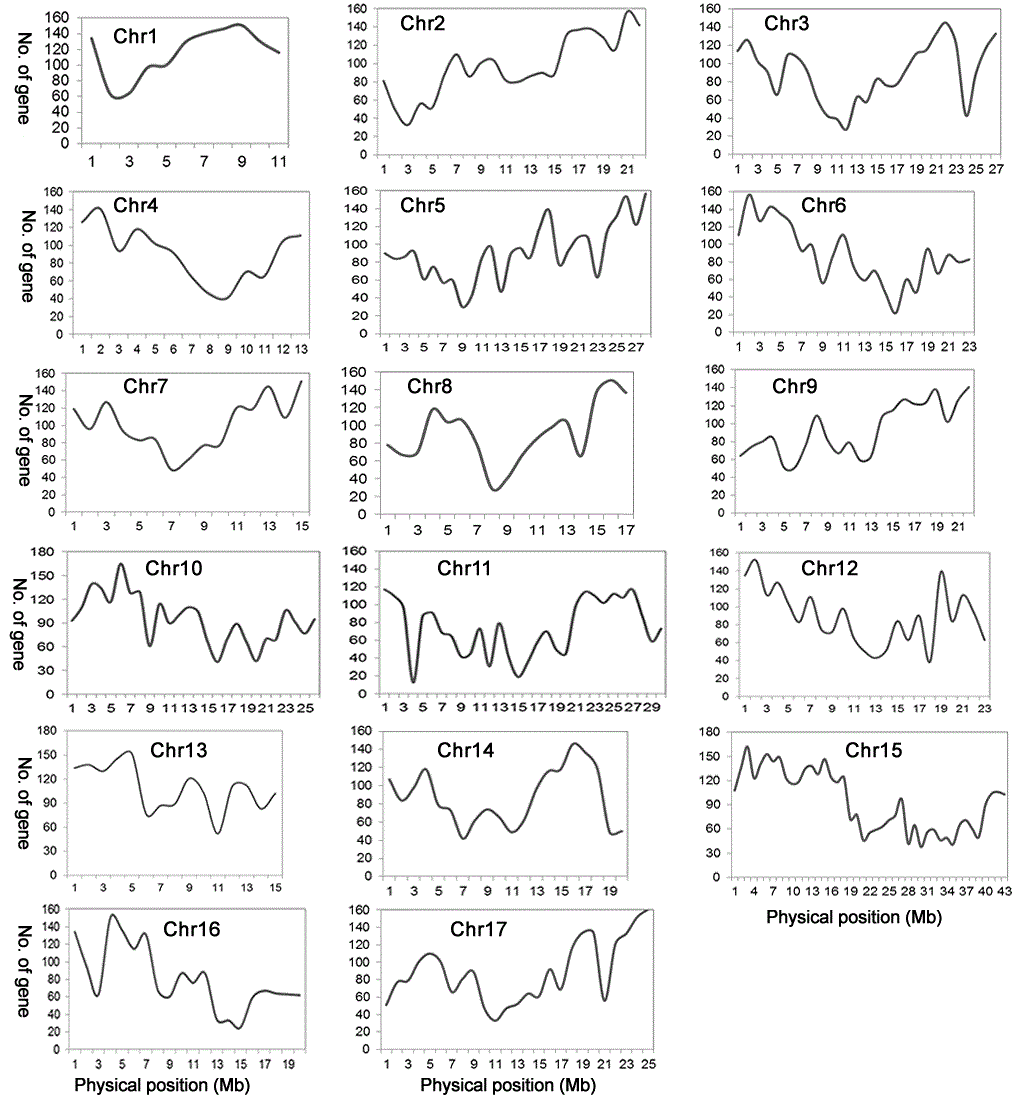
**

**Figure S4.** Distribution of anchored genes through 17 pear (*P. bretschneideri*) chromosomes (1-Mb window).


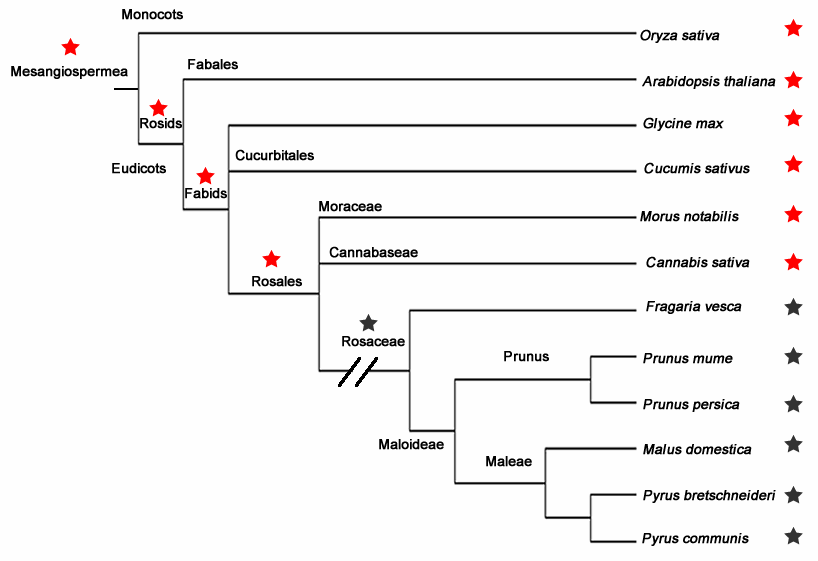


**Figure S5.** Taxonomy tree of 12 species.

The taxonomy tree was built using the common tree tool on the NCBI website (http://www.ncbi.nlm.nih.gov/Taxonomy/CommonTree/wwwcmt.cgi). The red star and black star represent harboring and loss of *Copia Maximus* lineage *env*-like protein, respectively.


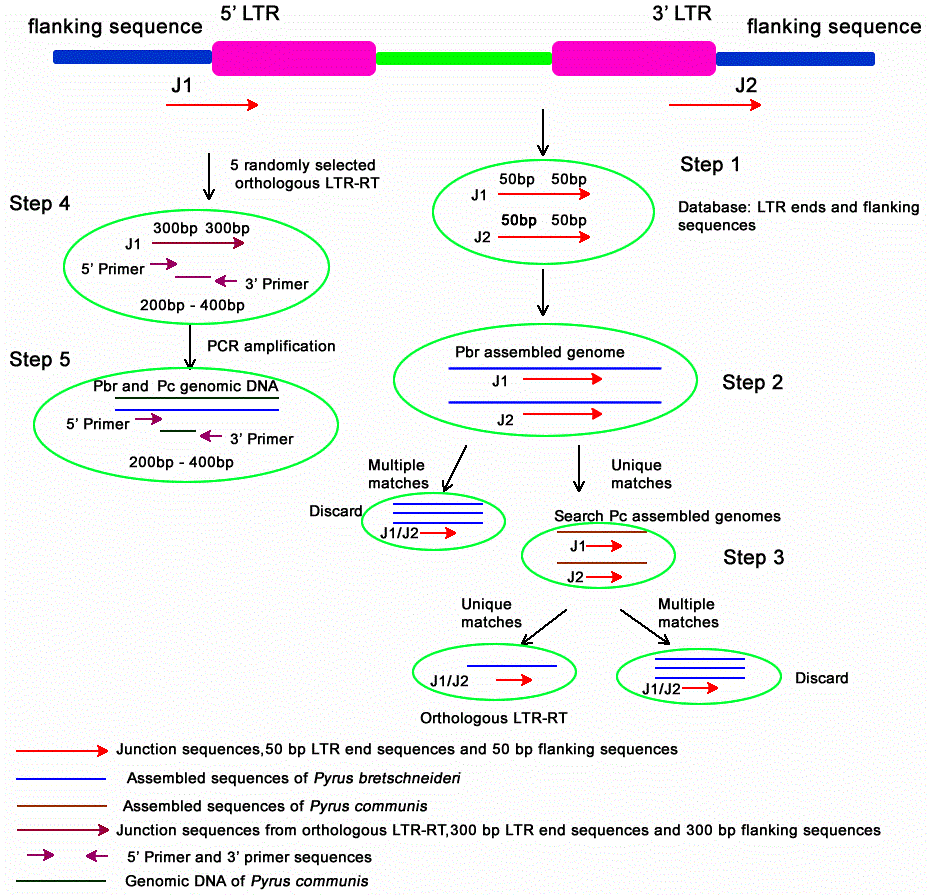


**Figure S6.** Pipeline of identification and verification of orthologous LTR-RTs between two *Pyrus* species


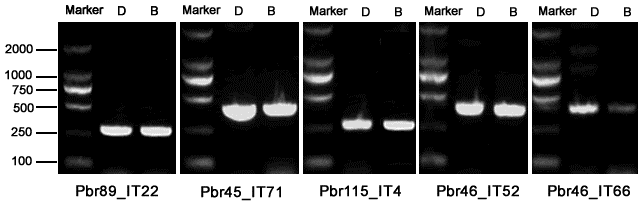


**Figure S7**. PCR-based validation of 5 orthologous LTR-RT elements in the two pear genomes.

D represent cultivar of ‘Dangshansuli’, B means cultivar of ‘Bartlett’


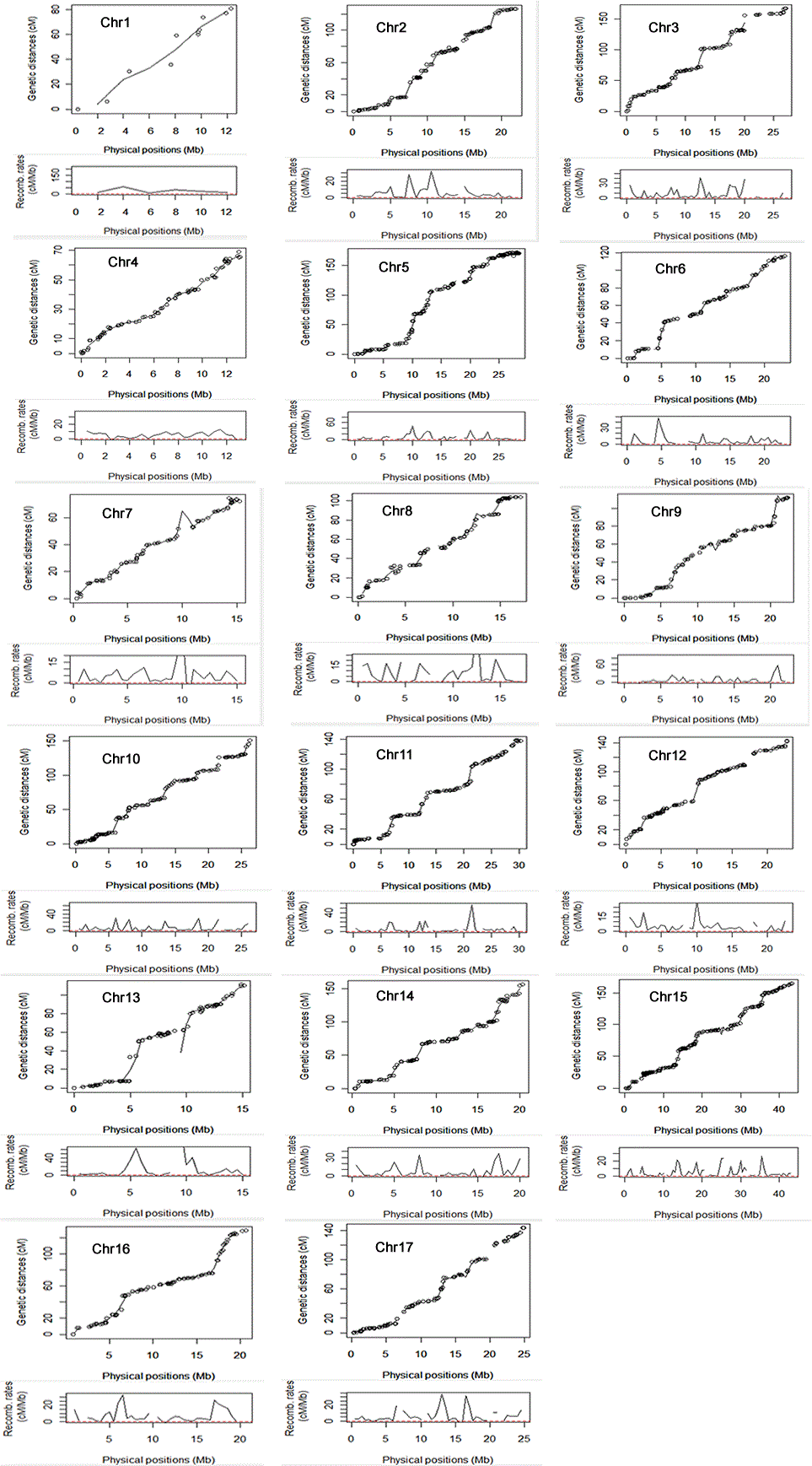


**Figure S8**. Genetic and physical maps of the 17 pear chromosomes and the estimated local GR rates.

For each chromosome, circles represent the genetic and physical positions of markers. The curves below the marker plots represent the estimated local GR rates.


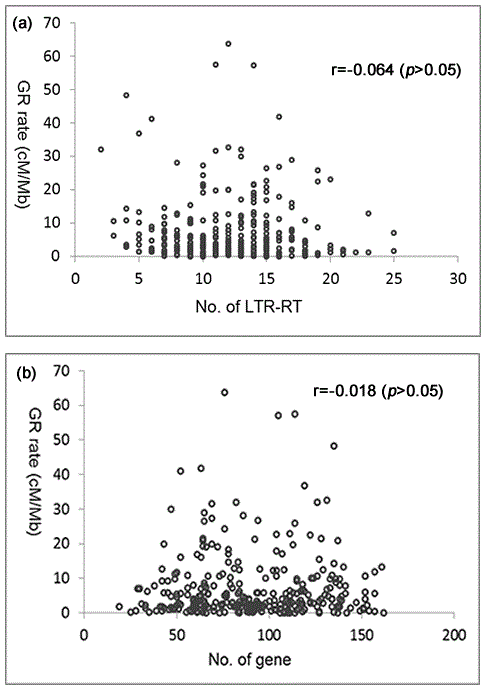


**Figure S9**. Correlations of genomic features with GR rates and gene densities.

1. LTR-RT densities (/Mb) plotted against GR rates; (b) Gene densities (/Mb) plotted against GR rates.


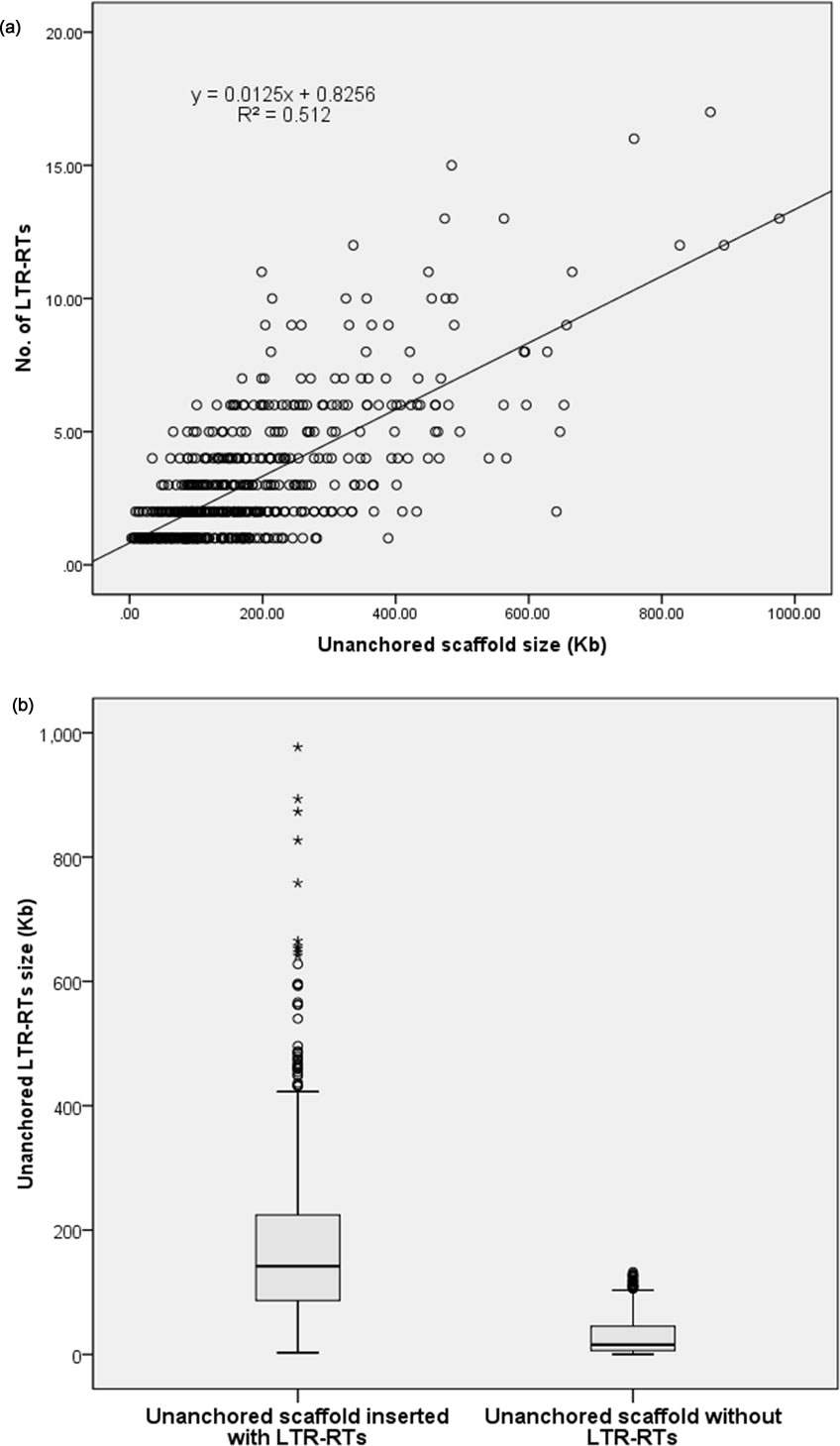


**Figure S10**. Comparison of unanchored scaffolds with LTR-RTs and unanchored scaffolds without LTR-RTs.

(a) Correlation between unanchored scaffolds size and the number of inserted LTR-RTs; (b) Boxplot comparisons of size of unanchored scaffolds with LTR-RTs and without LTR-RTs .
